# Emphasized Mechanistic Antimicrobial Study of Biofunctionalized Silver Nanoparticles on Model* Proteus mirabilis*

**DOI:** 10.1155/2018/3850139

**Published:** 2018-05-22

**Authors:** Asra Parveen, Manjunath S. Yalagatti, Venkataraman Abbaraju, Raghunandan Deshpande

**Affiliations:** ^1^H.K.E.S's Matoshree Taradevi Rampure Institute of Pharmaceutical Sciences, Gulbarga 585105, Karnataka, India; ^2^SriKrupa Institute of Pharmaceutical Sciences, Siddipet, Medak 502277, India; ^3^Materials Chemistry Laboratory, Department of Material Science, Gulbarga University, Gulbarga 585106, Karnataka, India

## Abstract

Antimicrobial study of biofunctionalized silver nanoparticles has been done with the emphasis on its mechanism on both gram positive and negative bacteria. The biofunctionalized silver nanoparticles are employed considering their importance in green chemistry with respect to easy synthesis, usefulness, and economic synthetic procedure involved. The stability of these nanoparticles was determined by zeta potential analyzer. The probable mechanism of antibacterial activity was performed on* Proteus mirabilis* by field emission scanning electron microscopy (FESEM) and energy dispersive spectroscopy (EDAX) study which does not show the presence of silver. The free radicals generated by silver nanoparticles were responsible for lethal antibacterial activity by rupturing the cell surface which causes improper nutrient and signal supply. Free radical scavenging efficacy of silver nanoparticles was confirmed by 1,1-Diphenyl-2-picrylhydrazyl (DPPH) method. AgNP enhanced the membrane leakage of reducing sugars by destroying the proteins existing on the cell wall. These nanoparticles are found to be toxic against human pathogens and are highly effective on* Staphylococcus aureus*. The effect of silver nanoparticles is concentration dependent and independent of the type of strains used.

## 1. Introduction

Biofunctionalized inorganic nanoparticles have attracted increasing attention in medical applications [1–3]. Silver (Ag) has been widely used inorganic antibacterial agent since ancient times to fight infections and control spoilage. The antibacterial and antiviral actions of Ag, Ag^+^ ions, and Ag compounds have been investigated to some detail [4, 5]. The Ag^+^ ions and salts of Ag have partial use as antimicrobial agents due to several reasons. The AgNP synthesized from pomegranate juice have shown shape and dose-dependent bactericidal effect on gram negative and gram positive bacteria [6]. The salts used in the antimicrobial mechanism continuously release Ag^+^ ions at a high concentration causing toxicity to the host. Such problems can be avoided by using functionalized silver nanoparticles (AgNP). AgNP have been used in antibacterial clothing and burn ointments and as a coating for medical devices, owing to their mutation-resistant antimicrobial activity [7–10].

AgNP can be synthesized using chemical, physical, and biological methods. The chemical and physical synthesis of nanoparticles built up flammable or harmful reagents and created a concern for their safe disposal [11–13]. The nanoparticles surface functionalization with chemical adsorbents may become unfit for medical application [14, 15]. Hence, efforts are being made to produce AgNP using a biological base like microorganisms and plant extracts. This procedure is quite attractive which yields highly biocompatible nanoparticles helpful in enhanced biodetection sensitivity. The use of plant parts is beneficial as compared to organisms as it is pathogenic, laborious, time-consuming, hygiene maintenance of cell culture that arises during large-scale production [16–20].

Hence, we have used the AgNP biosynthesized from guava leaf* (Psidium guajava)* aqueous extract for studying the antimicrobial effect and mechanism involved therein [21]. We have selected guava leaf as this plant is easily available and is used in many domestic and medicinal purposes as an antibacterial agent [22–24]. In our antimicrobial studies, the activity of AgNP was investigated against gram +ve and gram –ve microbes. Results infer that* Staphylococcus aureus* and* Providencia alcalifaciens* were inhibited at a very low concentration of AgNP. The growth-inhibitory effects on* Proteus mirabilis* and* Salmonella typhi* were moderate at higher concentration.

Many antimicrobial mechanisms proposed have reported that AgNP can easily be attached to the surface of the microbial cell membrane and disturb its permeability and respiration functions [25–28]. The effect of biofunctionalized and stable AgNP on antimicrobial mechanism has not been revealed clearly. Researchers have highlighted the antimicrobial mechanism as an effect of free radical generated by AgNP. The antimicrobial effect was investigated by electron spin resonance spectroscopy and reactive oxygen species (ROS) was understood to be the reason behind the possible mechanism [29]. In an interesting study, researchers' stated that biosynthesized AgNP and silver ions interrupted the mitochondrial activity by producing the intracellular ROS causing antiproliferative activity in HeLa cells [30].

## 2. Material and Method

The detailed study on microwave assisted extracellular biosynthesis of AgNP using aqueous guava leaf extracts* (Psidium guajava)* solution and aqueous AgNO_3_ solution reported in our earlier work. AgNP was roughly spherical in shape and in the range of 26 ± 5 nm in size indicating that they are disperse in nature [21].

### 2.1. Stability of Aqueous Colloidal Suspension of AgNP

The functionalized AgNP colloidal solution was stored for 30 weeks and analyzed for its stability. Periodically aliquot of the centrifuged reaction mixture was taken and subjected to UV-Vis spectroscopy measurements on an ECIL 5704SS UV-visible spectrophotometer at a resolution of 1 nm. The size and stability of the aqueous AgNP colloidal solution were reconfirmed by PALS zeta potential analyzer. The morphological changes of the bacterial cell surface were examined by the field emission scanning electron microscopy (FE-SEM, FEI Nova nano 600, Netherlands) and the images operated at 15 kV on a 0° tilt position.

### 2.2. *In Vitro *Pharmacological Activity

#### 2.2.1. Antibacterial Activity

The nutrient broth was prepared by dissolving peptone, 10 g; NaCl, 10 g; yeast extract, 5 g; and agar, 20 g in 1000 mL of distilled water. Initially, the stock cultures of* Staphylococcus aureus, Salmonella typhi, Providencia alcalifaciens,* and* Proteus mirabilis *were revived by inoculating in broth media and grown at 37°C for 18 h. The media were autoclaved and cooled to ~55°C. The required volume of test samples with different concentrations (2.5, 5, 10, and 20 *μ*g/mL) of AgNP were added and mixed well. The media were poured into the preautoclaved petri dishes. The 10^4^ CFU/ml culture was inoculated and grown at 37°C for 24 h. The control plates were prepared with blank (C_1_) and (C_2_) with guava leaf extract only. Different concentrations of (2.5, 5, 10, and 20 *μ*g/mL of AgNP) were used to study the antibacterial effect. The standard (silver sulfadiazine) plates were also used to study the antimicrobial effect for comparison purpose [31].

#### 2.2.2. Antimicrobial Mechanism

For understanding the antimicrobial mechanism,* Proteus mirabilis* was used as a model organism by Cup-plate method after 24 h. The plate was inoculated with* P. mirabilis* culture of 10^4^ CFU spread evenly and a well was punctured and filled with 20 *μ*g/mL functionalized AgNP solution (the highest concentration used in above antimicrobial studies). We strongly believe that the action of silver nanoparticles coordinates with ionic silver but the antimicrobial mechanism happens through free radical scavenging and by destructing the cell membrane. The plate was incubated at 37°C for 24 h and the diameter of zone of inhibition was visually observed. Field emission scanning electron microscope (FESEM) image of a healthy microorganism in the sample at different time interval was captured by collecting the sample from the internal periphery of zone of inhibition. The energy dispersive spectroscopy (EDAX) of the bacterial sample was also taken from the same place where the FESEM has been done. Free radical mechanism of AgNP from the zone of inhibition was studied and discussed separately. Here we have simulated the results of antibacterial effect and correlated with advanced microscopic and spectroscopic analytical techniques.

#### 2.2.3. Free Radical Scavenging Activity

Biosynthesized AgNP aqueous solution and butylated hydroxy anisole (BHA) were taken at different concentrations (2.5 *μ*g, 5 *μ*g, and 10 *μ*g/mL) in different test tubes. The volumes were adjusted to 1000 *μ*L by adding methanol. Five milliliters of 0.1 mM methanolic solution of 1,1-diphenyl-2-picryl hydrazyl (DPPH) was added to these tubes and shaken vigorously. The tubes were allowed to stand at 27°C for 20 min. The control was prepared without addition of AgNP. The absorbance of the functionalized AgNP was measured with UV-Vis spectroscopy at 517 nm. Radical scavenging activity was calculated using the following formula [32]:

(1) %  radical  scavenging  activity=  control  OD  −  aqu.  bio-AgNPs  ODcontrol  OD×100

#### 2.2.4. Estimation of Proteins and Reducing Sugars

The protein content of samples was determined by the Bradford method (1976). The reagents were prepared using 10 mg Coomassie Brilliant Blue G-250 dissolved in 0.5 ml of 95% ethanol. 10 ml of o-phosphoric acid was added to the solution and the volume was made up to 100 ml by adding distilled water. 5 ml of Bradford reagent was treated with 0.2 ml of extract and incubated for 5 min at room temperature. The color developed was measured at 595 nm. Extract control was prepared with Bradford reagent and used as blank. The protein content (per ml) with respect to time and AgNP concentration was determined with the help of standard Bovine Serum Albumin (BSA) standard curve. The estimation of reducing sugar was performed using the chromogenic DNSA method (Miller, 1959). Dinitrosalicylic acid (DNS) solution was prepared by dissolving 1 g of DNS in 2 N sodium hydroxide and 30 g of potassium sodium tartarate and the volume was made up to 100 ml by adding distilled water. 1.0 ml extract was mixed with 1 ml of DNS reagent and placed in boiling water bath for 5 min and cooled at room temperature and the absorbance was measured at 540 nm. The extract was used as blank while reading absorbance. The concentration of total reducing sugar was estimated with the help of standard maltose curve.

## 3. Results

**Figure 1:**
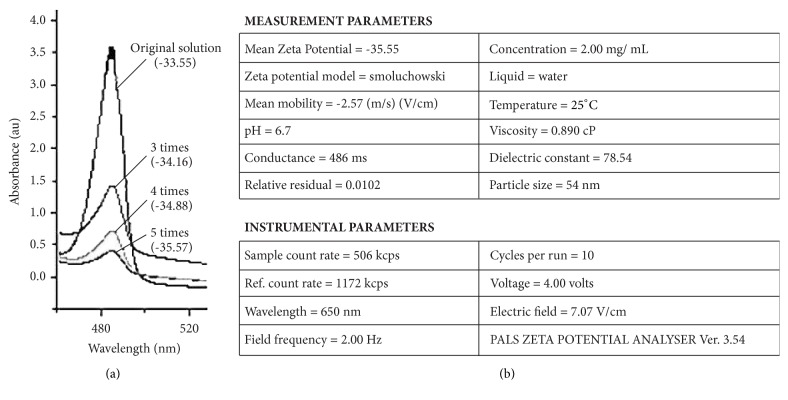
(a) Change in the absorbance of AgNP after the dilutions. (b) Change in zeta potential of AgNP after respective dilution and ultracentrifugation.

UV-Vis spectra analysis of AgNP and zeta potential biofunctionalized AgNP are shown in (Figures 1(a) and 1(b)). Zeta potential studies of 30-week stored guava functionalized AgNP aqueous solution showed the average particles size of 54 nm.

**Figure 2:**
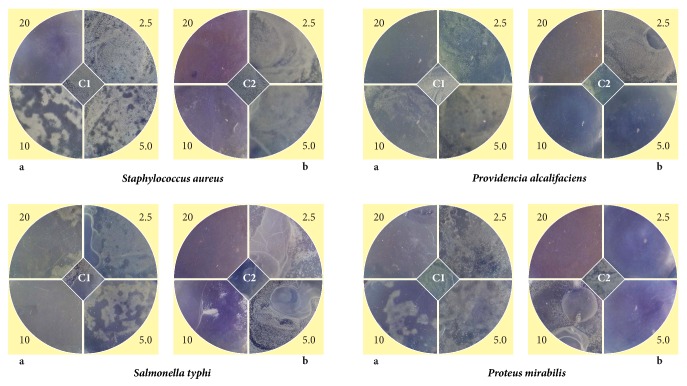
Antimicrobial activity of biofunctionalized AgNP using guava leaf solution. a and b represent the microbial plates treated with different concentrations of AgNP and silver sulfadiazine. The center portion marked as C1 shows the control and C2 guava leaf extract, respectively. All the concentrations were taken in *μ*g/mL.

Antimicrobial tests were performed against different microbes, namely,* Staphylococcus aureus, Salmonella typhi, Providencia alcalifaciens,* and* Proteus mirabilis *treated with different concentrations of AgNP (2.5 *μ*g to 20 *μ*g/mL) and silver sulfadiazine as standard (Figure 2).

**Figure 3:**
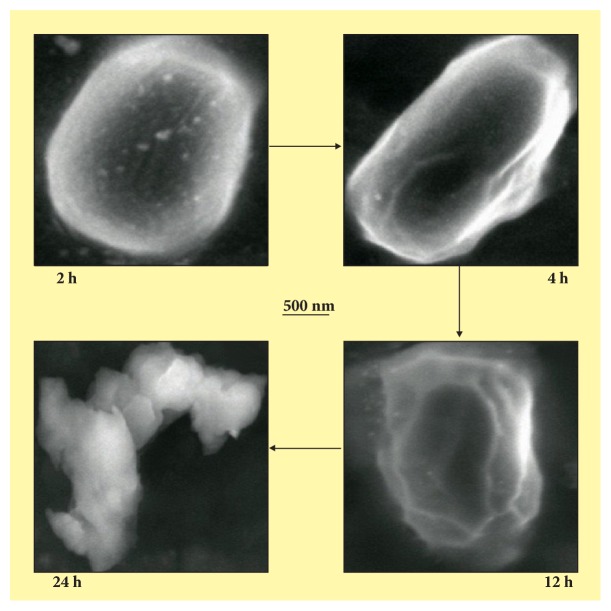
FESEM images taken with respect to time for the model microorganism* Proteus mirabilis *treated with functionalized AgNP for 24 h of incubation.

The control plates were prepared without addition of any drug for comparing the inhibitory effect of the standard silver sulfadiazine and the functionalized AgNP. The results revealed that the minimum inhibitory concentration (MIC) of AgNP against* S. typhi* was slightly higher at 10 *μ*g/ml where as in case of* P. mirabilis* MIC was found at 10 *μ*g/ml.* S. aureus and P. alcalifaciens* were found effective at 5 *μ*g (Table 1).  Figure 3 shows different FESEM images taken with respect to time for the model microorganism* Proteus mirabilis *treated with functionalized AgNP for 24 h of incubation.

**Figure 4:**
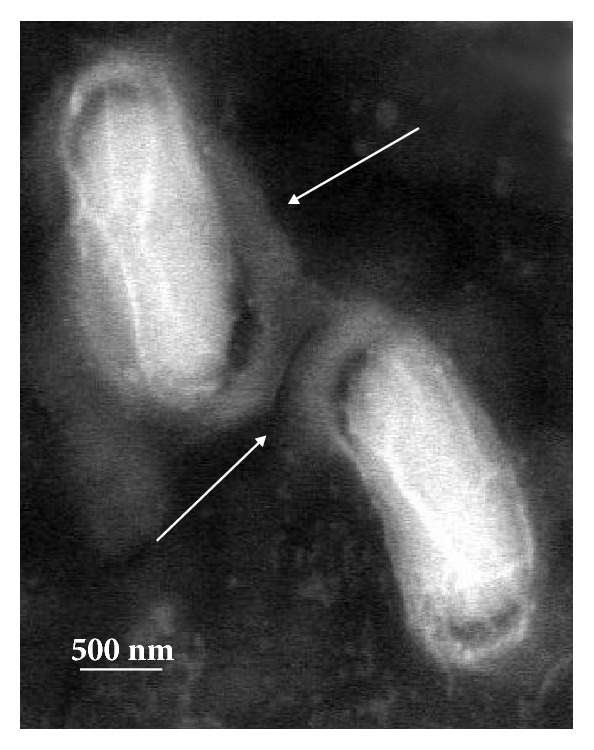
Arrows indicating the reduction in cell wall constituent causing destruction of the cell membrane's permeability.

AgNP were responsible for creating the lethal effect on the membrane of microorganisms. The cell was completely ruptured due to marked reduction in the cell wall constituents and hence AgNP destroys the permeability of the cell membranes (Figure 4).

**Figure 5:**
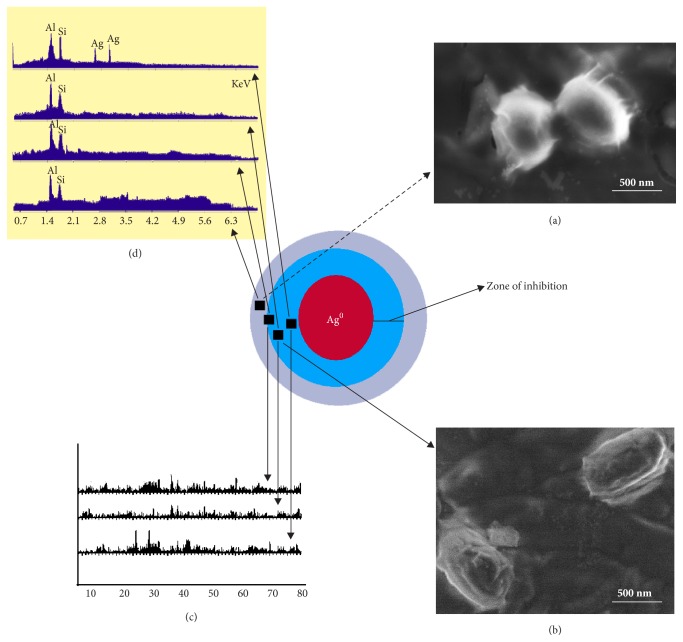
Antimicrobial study of AgNP against* P. mirabilis *with zone inhibition method. (a) and (b) are the FESEM images of the live and dying cells from the exact periphery of zone of inhibition. (c) and (d) are the XRD and EDAX graphs confirming the absence of Ag at the inhibition zone.

FESEM images of the live cell from the control plate and the ruptured dead cell were treated with AgNP from the exact edge of inhibition zone (Figures 5(a) and 5(b)). XRD and EDAX graphs confirm the absence of AgNP at the zone of inhibition (Figures 5(c) and 5(d)).

**Figure 6:**
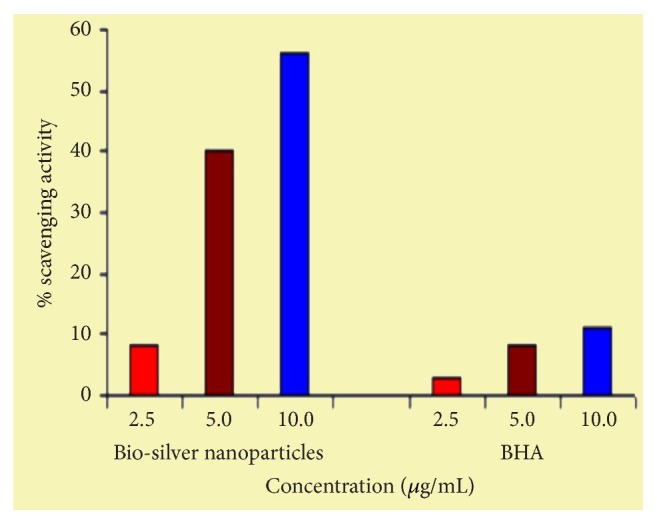
Free radical scavenging activity histogram of AgNP and BHA (butylated hydroxy anisole) shows the quenching efficacy of DPPH radical at different *μ*g/mL.

Arrows indicate the probable location of the samples picked up from the zone of inhibition. Radical scavenging efficacy of AgNP was performed using standard DPPH method to understand the antimicrobial mechanism.  Figure 6 shows the graphical presentation of scavenging the DPPH free radical in a concentration dependent manner.

Antibacterial assay of AgNP and silver sulfadiazine against the selected bacteria is shown in Table 1.

The amount of decreased proteins and increased reducing sugars in the cells treated with AgNP with respect to the time and concentration (Table 2).

## 4. Discussion

### 4.1. Stability of Colloidal AgNP Suspension

The biosynthesized AgNP solution was diluted for 5 times and preserved for 30 weeks before studying the stability of the solution. The dilution was carried out to bring the homogeneity in the solution and to get the absorbance value in UV-Vis absorbance under 1 nm. During dilution a proportionate hypochromic shift of surface plasmon was observed indicating no further changes in the shape, size, and interrelation between nanoparticles surface and biological moiety [21]. The UV peak of the dilute aqueous AgNP solution shows slight hypochromic shift attributed to the agglomeration of this AgNP in the original solution. This shows uniformity of biofunctionalized AgNP even after ultrasonication confirming the stability of colloidal solution. The zeta potential of the synthesized and preserved colloidal solution was −33.55 which improved to −35.57 after removal of sediment AgNP and extracellular organic impurities on ultrasonication. However, this was higher than that of microscopic images of fresh samples of AgNP solution reported using TEM and AFM in our earlier studies [21]. The increase in size may be attributed to the surface modification of AgNP with water soluble extracellular biomoieties of the guava leaf extract.

### 4.2. Antibacterial and Antioxidant Activity

**Figure 7:**
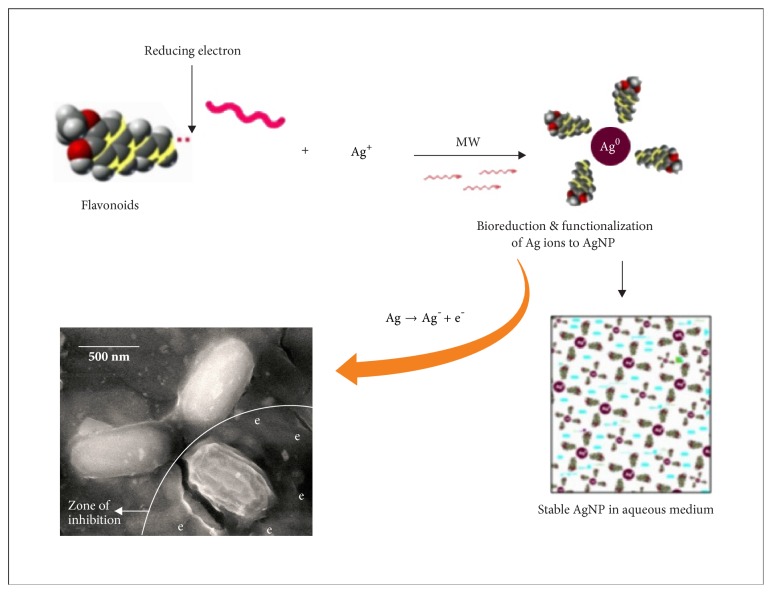
Microwave (MW) assisted rapid AgNP biosynthesis using guava leaf and its antibacterial screening. Microscopic image infers electrons release from AgNP free radical showing lethal effect on bacterial cell wall.

The negligible concentration used in the study evident that the functionalized AgNP with water soluble component of guava leaf extract has synergic effect in antibacterial activity. Comparing with the silver sulfadiazine, AgNP showed almost similar growth inhibition effect against* S. typhi* and significant growth inhibition from all other gram positive and negative microorganisms. AgNP showed a moderate growth-inhibitory effect against gram negative pathogen* S. typhi* at high concentration (>10 *μ*g). It has similar inhibitory effect compared with the standard drug silver sulfadiazine of same concentration. Control plates have not shown any antibacterial effect used in the present study (Figure 2). There was no antimicrobial activity observed in the solution devoid of AgNP in control plates as shown in the center (marked as “C_1_” for blank and “C_2_” containing the guava leaf extract). The study infers that the biofunctionalized AgNP using guava leaf extract showed satisfactory bactericidal activity at low concentration on different microbes and was independent of type of the strain used. In our results, biofunctionalized AgNP was found most effective against* S. aureus* and* P. alcalifaciens* (Figure 2 and Table 1). We have used gram negative* Proteus mirabilis* as a model organism to understand the antimicrobial mechanism as this bacterium found highly pathogenic and responsible for most of the* proteus* infections in humans with anatomical abnormalities, immunodeficiency, and long-term urinary catheterization [33–35]. AgNP was used to study the antimicrobial effect on* P. mirabilis* in application for the development of bacterial biofilms in patients with indwelling urinary catheters [36]. The other reason for selecting this microorganism was that the rate of antibacterial activity of the AgNP on* P. mirabilis *was slow as compared to other microorganisms and it becomes easy to understand the antibacterial mechanism. The MIC of AgNP against* S. aureus* was estimated to be lower than 5 *μ*g, and the growth inhibition effect was observed to be in a concentration dependent manner. The overall charge of bacterial cells at biological pH values was negative because of excess number of carboxylic groups, which upon dissociation makes the cell surface negative. The opposite charges of bacteria and nanoparticles attribute their adhesion and bioactivity due to electrostatic forces. Based on this theory, the binding of nanoparticles to the bacteria depends on the surface area available for interaction and the antibacterial mechanism of AgNP. However, nanoparticles having larger surface area enhance the bactericidal effect compared to the large sized particles; hence they impart cytotoxicity to the microorganisms [37]. The study also revealed that AgNP could enhance the membrane leakage of reducing sugars by destroying the proteins existing on the cell wall. Initially when the bacterial supernatant solid layer was exposed to plant extract has shown the high level of proteins with almost negligible leakage of reducing sugars. We observed the increased amount of reducing sugars but decrease in protein content from the treated bacterial cell. With increase in the concentration of AgNP there was gradual increase in leakage of reducing sugar with diminished concentration of proteins (Table 2). There was no release of reducing sugar detected from bacterial cells in control experiment. While the amount of reducing sugars released from cells treated with AgNP was 2.20 *μ*g per bacterial dry weight of 1 mg (mg/ml). The amount of reducing sugars was up to 93 *μ*g per bacterial dry weight of 1 mg after treating with AgNP for 24 h. There was only 30 *μ*g/mg of reducing sugar found in control experiment, suggesting that the AgNP accelerates (three times higher) the release of reducing sugars from bacterial cytoplasm. The highest amount of leakage of reducing sugars and proteins was observed from the intracellular cytosol after the treating* Halophilic archaea *with AgNP [38]. Research on the studies of synthesis and antimicrobial mechanism of functionalized AgNP was a continuous process. The clay functionalized AgNP have disrupted the membrane integrity and increased the production of intracellular ROS thereby increasing the energy dependent metabolism [39, 40]. The free radical efficiency of AgNP on ROS system and its charge discharge model have been reported [41, 42]. Hence we believe that the powerful oxidant evolves in the aqueous AgNP solution in the form of electrons and silver free radicals, which acts critically on the charged nuclear membrane of the bacterial cell wall. It is concluded that the free radicals and their electrons significantly increase the cell permeability. This affects the proper transport through plasma membrane, leaving the bacterial cells incapable of proper functioning resulting in cell death. We have clearly demonstrated FESEM images taken carefully at different time interval without disturbing the shape and integrity of the cell structure (Figure 3). We believe that the efficacy of DNA might have lost and cellular proteins were highly disturbed which are shown through critical changes and damage in the membrane structure. 10 *μ*g/mL of AgNP was sufficient to produce complete bacteriostatic effect on the bacterial colony of 10^4^ CFU/mL. FESEM image of the 10 *μ*g/mL AgNP treated* P. mirabilis* cell surface in liquid Mueller Hinton (MHA) medium after 12 h (Figure 4). The image clearly indicates that AgNP directly acts on the bacterial cell wall. Marked reduction in the cell wall constituents was observed by affecting the permeability of the cell membranes. At higher concentration of AgNP the bacteria get scattered and isolated. The membrane looks severely damaged, and the components become disorganized. Hence, MIC/MBC levels of the functionalized AgNP were found to be lower than standard drug silver sulfadiazine which acts by producing Ag^+^ ions. Comparative FESEM image of* P. mirabilis *healthy cell and AgNP treated cell was picked after 12 h exactly from the zone of inhibition (Figures 5(a) and 5(b)). The image shows severe damage to the bacterial cell surface by presence of wrinkles and all the intracellular contents were leaked (shown with arrow mark). XRD and EDAX images revealed absence of AgNP suggesting that there was no direct attachment of nanoparticles to the cell surface causing rupture of the membrane or the Ag^+^ ions make any influence for the antimicrobial activity (Figures 5(c) and 5(d)). EDAX shows “Al” peak which was of the substrate base used for the FESEM scanning and “Si” the traces of glass plate used for antibacterial studies. High care was taken while doing the XRD and EDAX experiment so that the microbes should not get affected with low vacuum environment. Hence AgNP has generated free radicals which proved lethal to the cell surface by acting on the unsaturated lipid molecules of the cell membrane which was easily susceptible for the damage. These free radicals attack the nearest healthy cell and steal its electron. When cell loses its electrons it becomes a free radical and begins a chain reaction. When this cascade of reaction was initiated on the surface of the cell wall, the total disruption of a living cell was seen within a short time (Figure 7). This progression would continue to go on from cell-to-cell and bacteria-to-bacteria causing electron propagation all around the flora. With greater surface area to volume ratio of smaller AgNP, more Ag atoms get in contact with the solution than the larger nanoparticles. Smaller size of the nanoparticles has higher probability of cell membrane destruction [43]. The surface of AgNP was responsible for the release of free radicals and electrons. The large size nanoparticles produce less free radical than smaller size nanoparticles available in high quantity. We strongly believe that these free radicals impart antibacterial properties. AgNP shows concentration and size dependent antibacterial effect. The free radical electrons produced along with the formation of free radicals of AgNP were responsible for producing lethal effect on the membrane of microorganisms. This results in shrinkage and rupture of the cell surface and improper nutrient and signal supply. The increased concentration of AgNP generates more free radical electrons thereby enhancing the surface area. The dose dependent antimicrobial effect may be attributed to this phenomenon [44, 45]. There was no possibility of accumulation of Ag^+^ ions in the host; it may be negligible if found in traces. Radical scavenging efficacy of AgNP was studied using standard DPPH method to confirm the generation of free radicals in antimicrobial mechanism [46]. Scavenging the DPPH free radical in a concentration dependent manner can be seen (Figure 6). AgNP scavenged the DPPH free radical five times more effectively than butyl hydroxy anisole (BHA). Even at low concentration of 5 *μ*g where BHA had less than 10% efficiency, biofunctionalized AgNP mopped up more than 40% free radical* in vitro*. Similarly the percentage of quenching effect on DPPH free radical was 9% with 2.5 *μ*g of AgNP, where BHA shows only 3% at the same minimum concentration used. AgNP and BHA scavenged 56% and 12%, respectively, at a maximum concentration of 10 *μ*g/ml. Statistical analysis (test of significance) of the data obtained from the free radical scavenging activity of AgNP using *T*-test indicated that the difference on the DPPH free radical used was more significant (*P* < 0.01) than BHA.* In vitro* assays of DPPH radical scavenging and ABTS radical scavenging have exhibited antioxidant potential of synthesized AgNP.

In the current research work, we tried to elucidate the antimicrobial mechanism of AgNP satisfactorily but still there are many enigmas to be solved with respect to bacterial resistance, biocompatibility, and interactions with other drugs. In conclusion, establishing safety and efficiency of biofunctionalized AgNP requires substantial preclinical and clinical studies.

## 5. Conclusion

Biofunctionalized silver nanoparticles were prepared by economical and eco-friendly green method. The free radicals and its electrons were lethal and significantly increase the permeability of the cell wall and disturb the proper transport through plasma membrane. Thus the phenomenon leads to improper functioning of the bacterial cells which further results in cell death. The accumulation of AgNP on the surface of the cell wall was negligible and hence will be quite safer compared to other silver salts as it avoids host cell-nanoparticles interaction. The study encourages safer use of biofunctionalized AgNP for antibacterial, drug delivery, and wound healing applications.

## Figures and Tables

**Table 1:** MIC and MBC of biofunctionalized AgNP and silver sulfadiazine in *μ*g/mL.

Compound	*S. aureus*		* S. typhi*		*P. alcalifaciens*		*P. mirabilis*	
	MIC	MBC	MIC	MBC	MIC	MBC	MIC	MBC
AgNP	<5	2.5	>10	5	5	2.5	10	5
Silver sulfadiazine	<10	5	>10	5	20	>10	<20	>5

*Note*. MIC = minimum inhibitory concentration and MBC = minimum bacteriostatic concentration.

**Table 2:** Effect of time and concentrations of AgNPs on *P. Mirabilis* during antibacterial assay showing decrease in proteins and increase in reducing agents.

Influence of time			Influence of concentration		
Sample	Concentration of protein (mg/ml)	Concentration of sugars (*μ*g/ml)	Sample (*μ*g/ml)	Concentration of protein (mg/ml)	Concentration of sugars (*μ*g/ml)
0 hr	9.995	04	Control	4.576	30
2 hr	8.011	10	Extract	9.899	03
4 hr	7.667	22	2.5	5.249	22
8 hr	5.978	41	05	3.876	23
12 hr	4.011	56	10	3.112	40
18 hr	1.004	88	15	2.555	55
24 hr	0.005	93	20	2.202	76

## Supplementary Materials

Supplementary Materials Graphical abstract: microwave-assisted rapid AgNP biosynthesis using guava leaf and its antibacterial screening. Microscopic, spectroscopic techniques infer electrons release from AgNP free radical showing lethal effect on bacterial cell wall.

## References

[1] Schwenzer B., Scharnweber D. (2010). Biological nano-functionalization of titanium-based biomaterial surfaces: A flexible toolbox. *Journal of the Royal Society Interface*.

[2] Vladkova T. G. (2010). Surface engineered polymeric biomaterials with improved biocontact properties. *International Journal of Polymer Science*.

[3] Carrara S. (2010). Nano-bio-technology and sensing chips: New systems for detection in personalized therapies and cell biology. *Sensors*.

[4] Karunakaran G., Jagathambal M., Gusev A. (2016). Nitrobacter sp. extract mediated biosynthesis of Ag2O NPs with excellent antioxidant and antibacterial potential for biomedical application. *IET Nanobiotechnology*.

[5] Pal S., Tak Y. K., Song J. M. (2007). Does the antibacterial activity of silver nanoparticles depend on the shape of the nanoparticle? A study of the gram-negative bacterium *Escherichia coli*. *Applied and Environmental Microbiology*.

[6] Narducci D. (2007). An Introduction to Nanotechnologies: What’s in it for Us?. *Veterinary Research Communications*.

[7] Roy E., Patra S., Saha S., Madhuri R., Sharma P. K. (2015). Shape-specific silver nanoparticles prepared by microwave-assisted green synthesis using pomegranate juice for bacterial inactivation and removal. *RSC Advances*.

[8] Rai M. K., Deshmukh S. D., Ingle A. P., Gade A. K. (2012). Silver nanoparticles: the powerful nanoweapon against multidrug-resistant bacteria. *Journal of Applied Microbiology*.

[9] Peiris M. K., Gunasekara C. P., Jayaweera P. M., Arachchi N. D. H., Fernando N. (2017). Biosynthesized silver nanoparticles: Are they effective antimicrobials?. *Memórias do Instituto Oswaldo Cruz*.

[10] Singh H., Du J., Yi T.-H. (2017). Kinneretia THG-SQI4 mediated biosynthesis of silver nanoparticles and its antimicrobial efficacy. *Artificial Cells, Nanomedicine and Biotechnology*.

[11] Marcato P. D., Duran N. (2011). *Metal Nanoparticles in Microbiology*.

[12] Iravani S., Korbekandi H., Mirmohammadi S. V., Zolfaghari B. (2014). Synthesis of silver nanoparticles: chemical, physical and biological methods. *Research in Pharmaceutical Sciences*.

[13] Leung T. C.-Y., Wong C. K., Xie Y. (2011). Green synthesis of silver nanoparticles using biopolymers, carboxymethylated-curdlan and fucoidan. *Materials Chemistry and Physics*.

[14] Kemp M. M., Kumar A., Mousa S. (2009). Synthesis of gold and silver nanoparticles stabilized with glycosaminoglycans having distinctive biological activities. *Biomacromolecules*.

[15] Prabhu S., Poulose E. K. (2012). Silver nanoparticles: mechanism of antimicrobial action, synthesis, medical applications, and toxicity effects. *International Nano Letters*.

[16] Graf P., Mantion A., Foelske A. (2009). Peptide-coated silver nanoparticles: Synthesis, surface chemistry, and ph-triggered, reversible assembly into particle assemblies. *Chemistry - A European Journal*.

[17] Philip D., Unni C., Aromal S. A., Vidhu V. K. (2011). *Murraya Koenigii* leaf-assisted rapid green synthesis of silver and gold nanoparticles. *Spectrochimica Acta Part A: Molecular and Biomolecular Spectroscopy*.

[18] Railean-Plugaru V., Pomastowski P., Wypij M. (2016). Study of silver nanoparticles synthesized by acidophilic strain of Actinobacteria isolated from the of Picea sitchensis forest soil. *Journal of Applied Microbiology*.

[19] Mukherjee P., Ahmad A., Mandal D. (2001). Fungus-mediated synthesis of silver nanoparticles and their immobilization in the mycelial matrix: a novel biological approach to nanoparticle synthesis. *Nano Letters*.

[20] Jung W. K., Koo H. C., Kim K. W., Shin S., Kim S. H., Park Y. H. (2008). Antibacterial activity and mechanism of action of the silver ion in *Staphylococcus aureus* and *Escherichia coli*. *Applied and Environmental Microbiology*.

[21] Padalia H., Moteriya P., Chanda S. (2015). Green synthesis of silver nanoparticles from marigold flower and its synergistic antimicrobial potential. *Arabian Journal of Chemistry*.

[22] Raghunandan D., Mahesh B. D., Basavaraja S., Balaji S. D., Manjunath S. Y., Venkataraman A. (2011). Microwave-assisted rapid extracellular synthesis of stable bio-functionalized silver nanoparticles from guava (Psidium guajava) leaf extract. *Journal of Nanoparticle Research*.

[23] Deguchi Y., Miyazaki K. (2010). Anti-hyperglycemic and anti-hyperlipidemic effects of guava leaf extract. *Nutrition & Metabolism*.

[24] Jaiarj P., Khoohaswan P., Wongkrajang Y. (1999). Anticough and antimicrobial activities of *Psidium guajava* Linn. leaf extract. *Journal of Ethnopharmacology*.

[25] Abreu P. R., Almeida M. C., Bernardo R. M. (2006). Guava extract (Psidium guajava) alters the labelling of blood constituents with technetium-99m. *Journal of Zhejiang University Science B*.

[26] Velusamy P., Su C.-H., Kumar G. V. (2016). Biopolymers regulate silver nanoparticle under microwave irradiation for effective antibacterial and antibiofilm activities. *PLoS ONE*.

[27] Sharma V. K., Yngard R. A., Lin Y. (2009). Silver nanoparticles: Green synthesis and their antimicrobial activities. *Advances in Colloid and Interface Science*.

[28] Raffi M., Hussain F., Bhatti T. M., Akhter J. I., Hameed A., Hasan M. M. (2008). Antibacterial characterization of silver nanoparticles against *E.coli* ATCC-15224. *Journal of Materials Science and Technology*.

[29] Santos K. S., Barbosa A. M., Da Costa L. P., Pinheiro M. S., Oliveira M. B. P. P., Ferreira Padilha F. (2016). Silver nanocomposite biosynthesis: Antibacterial activity against multidrug-resistant strains of Pseudomonas aeruginosa and Acinetobacter baumannii. *Molecules*.

[30] Su H.-L., Chou C.-C., Hung D.-J. (2009). The disruption of bacterial membrane integrity through ROS generation induced by nanohybrids of silver and clay. *Biomaterials*.

[31] Gorbe M., Bhat R., Aznar E. (2016). Rapid Biosynthesis of Silver Nanoparticles Using Pepino (Solanum muricatum) Leaf Extract and Their Cytotoxicity on HeLa Cells. *Materials *.

[32] Nanda A., Saravanan M. (2009). Biosynthesis of silver nanoparticles from Staphylococcus aureus and its antimicrobial activity against MRSA and MRSE. *Nanomedicine: Nanotechnology, Biology and Medicine*.

[33] Patel A., Patel N. M. (2010). Determination of polyphenols and free radical scavenging activity of Tephrosia purpurea linn leaves (Leguminosae). *Pharmacognosy Research*.

[34] Jacobsen S. M., Stickler D. J., Mobley H. L. T., Shirtliff M. E. (2008). Complicated catheter-associated urinary tract infections due to *Escherichia coli* and *Proteus mirabilis*. *Clinical Microbiology Reviews*.

[35] Macleod S. M., Stickler D. J. (2007). Species interactions in mixed-community crystalline biofilms on urinary catheters. *Journal of Medical Microbiology*.

[36] Stickler D. J. (2008). Bacterial biofilms in patients with indwelling urinary catheters. *Nature Clinical Practice Urology*.

[37] Senior B. W. (1983). Proteus morgani is less frequently associated with urinary tract infections than Proteus mirabilis: An explanation. *Journal of Medical Microbiology*.

[38] Kim J. S., Kuk E., Yu K. N. (2007). Antimicrobial effects of silver nanoparticles. *Nanomedicine: Nanotechnology, Biology and Medicine*.

[39] Thombre R. S., Shinde V., Thaiparambil E., Zende S., Mehta S. (2016). Antimicrobial activity and mechanism of inhibition of silver nanoparticles against extreme halophilic archaea. *Frontiers in Microbiology*.

[40] He D., Jones A. M., Garg S., Pham A. N., Waite T. D. (2011). Silver nanoparticle-reactive oxygen species interactions: application of a charging-discharging model. *The Journal of Physical Chemistry C*.

[41] Patrascu M. E. B., Badea N., Pirvu C. (2016). Multifunctional soft hybrid bio-platforms based on nano-silver and natural compounds. *Materials Science and Engineering C: Materials for Biological Applications*.

[42] Jones A. M., Garg S., He D., Pham A. N., Waite T. D. (2011). Superoxide-mediated formation and charging of silver nanoparticles. *Environmental Science & Technology*.

[43] Kumari M., Pandey S., Giri V. P. (2017). Tailoring shape and size of biogenic silver nanoparticles to enhance antimicrobial efficacy against MDR bacteria. *Microbial Pathogenesis*.

[44] Zhu C., Xue J., He J. (2009). Controlled in-situ synthesis of silver nanoparticles in natural cellulose fibers toward highly efficient antimicrobial materials. *Journal of Nanoscience and Nanotechnology*.

[45] Yin H. (2007). Quest for better antioxidants: A commentary on “Enhanced radical-scavenging activity by antioxidant-functionalized gold nanoparticles: A novel inspiration for development of new artificial antioxidant”. *Free Radical Biology & Medicine*.

[46] Patra J. K., Baek K.-H. (2017). Antibacterial activity and synergistic antibacterial potential of biosynthesized silver nanoparticles against foodborne pathogenic bacteria along with its anticandidal and antioxidant effects. *Frontiers in Microbiology*.

