# Supplementary material for: Emphasized Mechanistic Antimicrobial Study of Biofunctionalized Silver Nanoparticles on Model Proteus mirabilis
Source: J Drug Deliv. 2018 May 22;2018:3850139. doi: 10.1155/2018/3850139 (PMC5987338; doi:10.1155/2018/3850139)
Supplement: Supplementary Materials — Graphical abstract: microwave-assisted rapid AgNP biosynthesis using guava leaf and its antibacterial screening. Microscopic, spectroscopic techniques infer electrons release from AgNP free radical showing lethal effect on bacterial cell wall. [file 3850139.f1.docx]

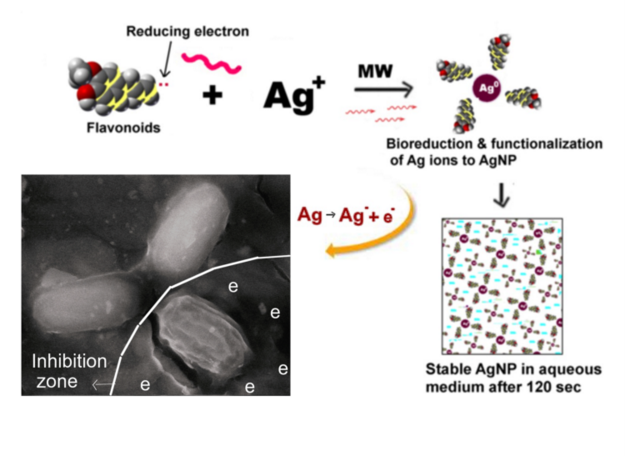


**Graphical Abstract:** Microwave-assisted rapid AgNP biosynthesis using guava leaf and its antibacterial screening. Microscopic, spectroscopic techniques infer electrons release from AgNP free radical showing lethal effect on bacterial cell wall.
